# Bright's Disease, Malaria, and Machine Politics: The Story of the Illness of President Chester A. Arthur

**DOI:** 10.1055/s-0037-1612632

**Published:** 2017-12-19

**Authors:** Theodore N. Pappas

**Affiliations:** 1 Division of Advanced Oncologic and Gastrointestinal Surgery, Duke University, School of Medicine, Durham, North Carolina

**Keywords:** Chester A. Arthur, Bright's disease, malaria, renal failure

## Abstract

In July of 1881, President James A. Garfield was shot in the back at the Sixth Street Train Station in Washington, D.C. Garfield died after an extended illness and Chester A. Arthur assumed the presidency on September 20, 1881. He served the remaining three and a half years but was ill for most of his term. Arthur died of the complications of Bright's disease less than two years after leaving office. In the 1880s, Bright's disease was the syndrome that described renal failure associated with proteinuria, but the etiology of Arthur's kidney failure has never been determined. Arthur is one of our least understood Presidents, owing to his brief tenure in office, his death shortly after leaving office, and the fact that he burned all his personal papers just prior to his death. This manuscript will explore the medical history of Chester A. Arthur, including his presumed diagnosis of malaria, his symptoms during his declining health, and will define the differential diagnosis of the causes of his renal failure that culminated in his death in November of 1886.

On July 2, 1881, Charles Guiteau shot President James A. Garfield in the back at the Sixth Street Station of the Baltimore and Potomac Railroad in Washington, D.C. The gunman shouted, “…I am a Stalwart and Arthur will be President…” after the shooting; announcing the controversial presidency of Chester A. Arthur. Garfield died two and one half months later of a delayed complication of his gunshot wound to the back. 1 Arthur assumed the presidency on September 20, 1881 and served the remaining three and a half years but was ill for most of his term. Arthur died of the complications of Bright's disease less than two years after leaving office. In the 1880s, Bright's disease was the syndrome that described renal failure heralded by proteinuria, but the etiology of Arthur's kidney failure has never been determined. Chester A. Arthur ( Fig. 1 ) is one of our least understood Presidents, owing to his brief tenure in office, his death shortly after leaving office, and the fact that he burned all his personal papers just prior to his death. This manuscript will explore the medical history of Chester A. Arthur, his symptoms during his declining health, and will define the differential diagnosis of the causes of his renal failure that culminated in his death on November 18 of 1886.

**Fig. 1:**
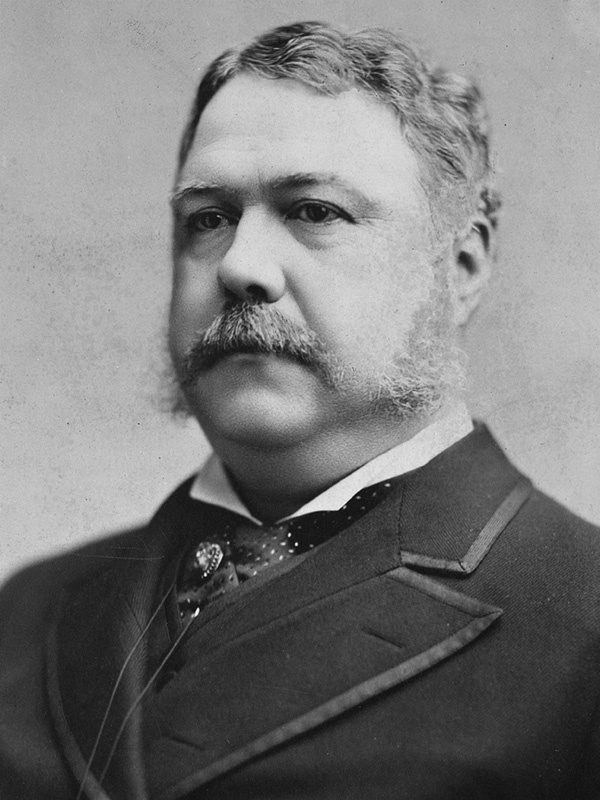
Chester A. Arthur, c1881.

## Early Life and Health

Chester A. Arthur was born on October 5, 1829 in Fairfield, Vermont. 2 He and his eight siblings were raised by their father (an ordained minister and teacher) and mother in several cities across the eastern United States. Arthur appeared to be a healthy child and young adult. There is no evidence of other illnesses in his family that would have predisposed Arthur to renal failure in later life.

Graduating from Union College in Schenectady, New York, in 1848, Arthur spent time as a schoolteacher before moving to New York City where he began “reading the law.” He was admitted to the bar in 1854 and practiced law in New York until he was elected to the Vice Presidency in 1880. 3 His law practice was temporarily interrupted by the Civil War when Arthur served as quartermaster for the New York regiments attaining the rank of Brigadier General. 4 As a young adult, Arthur was thought to measure ∼6 feet 2 inches tall and weigh ∼180 lbs. As a successful lawyer in New York, he lived a busy social life and gained the reputation for lavish eating and drinking when his weight swelled to 220 lbs. Still by examining several photographs of him as an adult, he would not be described as obese and had an approximate body mass index (BMI) of 28. 5

Through his political connections in the Ulysses S. Grant administration, Arthur became the Collector of the Port of New York at the New York Customs House. This position paid a hansom salary ($6,500 per year) but had a much larger earning power ($50,000 per year) due to the percentage of fines that were retained by the Collector. Arthur eventually gained a reputation as the worst example of the patronage system that was prevalent in this political era. Appointments to the Port Authority and every other civil servant position were made by patronage. Arthur and others in politically appointed positions often gained great wealth by these positions while at the same time were often ill suited in training or experience to hold these positions. Arthur was immensely successful in his position at the New York Custom House because he was a talented organizer (likely due to his quartermaster experience in the Civil War). Unfortunately, he was widely regarded as dishonest in his management of the port fines and the subsequent financial gains for his cronies. Arthur's relationship with The Rutherford B. Hayes Administration was soured by the fact that Hayes was determined to reform the patronage system and demanded the resignation of Arthur in 1878. Arthur's national reputation was tarnished by his dismissal from this position, a fact that would follow him through the rest of his political career. 6

Arthur was married to Ellen Herndon in 1859, and they had three children. The oldest died as a child in 1863 from a seizure disorder during what appeared to be an acute illness that could have been meningitis. 7 The symptoms of Arthur's renal failure probably did not start until 1880 or 1881, so it is unlikely that Arthur's illness was related to that which contributed to the death of his oldest son. Arthur's wife died of pneumonia on January 12, 1880 at age 42. She had come down with a cold and died a few days later. 8 Although it was common in the 1880s for individuals to succumb to pneumonia, the rapidity of her death after such a short prodrome suggests an aggressive bacterial illness such as a streptococcal infection. 9 There is no record that Chester Arthur contacted any illness from his dying wife but he clearly was emotionally affected throughout the rest of his life.

## Presidential Campaign of 1880

The Republican Party was split going into the nominating convention in Chicago in June of 1880. The Stalwarts division of the party, led by Roscoe Conkling, Senator from New York, and Arthur (proponents of patronage), and the Half Breeds (party division supporting modest civil service reform) were deadlocked for much of the convention. The Stalwarts backed former president Ulysses S. Grant, while the Half Breeds supported James G. Blane of Maine. The convention struggled to reach consensus after many ballots but the ultimate candidate to receive the majority of the votes was James A. Garfield of Ohio. Since Garfield was neither a Stalwart nor a Half-Breed, the convention chose to “balance the ticket” by nominating Chester A. Arthur as the vice presidential candidate. 10

The presidential election of 1880 was decided in favor of the Garfield/Arthur ticket by just over 2,000 votes out of over 8.8 million votes cast. The Democratic ticket that included General Winfield Scott Hancock and William H. English of Indiana was considered a strong pairing and the total vote count represented 78% of the eligible voters. Arthur played a significant role in the campaign by raising money in his home state of New York and was vigorous in his efforts to support the Republican ticket. The Republicans carried New York and with it its 35 electoral votes which provided the margin for victory in the Electoral College (Republicans 214 electoral votes, Democrats 155 electoral votes). 11

## Vice President Arthur

From March 4 until September 19 of 1881, Arthur served as Vice President and attempted to convince President Garfield that the Stalwart friends of the Vice President should be appointed to several cabinet posts. Garfield resisted and a battle ensued between Garfield and the Stalwarts in the U.S. Senate that opposed the President's nominations. When Garfield was shot on July 2, Arthur remained as a disempowered Vice President despite the fact that the after effects of the attempted assassination had incapacitated the President. Garfield died on September 19 after rupturing a pseudoaneurysm of the splenic artery, a direct sequela of the injuries sustained on July 2. 1 Arthur was in New York City and received the news of the President's death with great solemnity. He was sworn in just after 2 a.m. on the morning for the 20th in his New York townhome. 12

## President Chester A. Arthur

Arthur struggled in the first several months of his presidency as many members of the Garfield-appointed cabinet resigned. Arthur replaced these cabinet members with Stalwarts but eventually began balancing his appointments as he assigned politicians that were more progressive to key positions. Although his presidency was not noted for dramatic legislative innovation, he was responsible for modernization of the U.S. Navy and his positions on civil rights, immigration, and tariffs predicted future U.S. policy in these areas.

Early in his presidency, Arthur began experiencing fatigue. He was known as a vigorous entertainer, staying up late most nights and eating and drinking without restraint, but as his presidency progressed, he became more reserved. He still prided himself on entertaining in the White House and going on long walks after midnight in Washington, D.C. He rarely conducted presidential business before 10 a.m. and even more rarely after 4 p.m. Although his short business hours were often blamed on a lack of work ethic, 13 it is very likely that the President's illness was the driving factor behind his perceived lack of effort. Dr. Brodie Herndon, Arthur's brother-in-law, recorded the first evidence of the President's deteriorating health in February of 1882. Early in 1882, Herndon was invited to stay in the White House, and in his diary he noted that the President was “sick in body and soul.” A diagnosis was not attached to this quote so Herndon did not specify the cause of the President's illness. 14

There were news reports in 1882 that the President had contacted malaria. On September 4, the Chicago Tribune reported that Arthur and three other inhabitants of the white house staff had developed fever associated with symptoms of malaria. 15 The geography of Washington, D.C. was originally a very large swamp and the mosquito infestation in the D.C. area was notorious. Malaria was endemic in the nation's capital and in other southern coastal cities in the 1800s. 16 Over the subsequent century, urban expansion of Washington, D.C. eradicated the swamp surrounding the city ameliorating the mosquito and malaria problems. By October of 1882, Arthur's malaria seemed to be improving evidenced by his appearance on a fishing vacation where he was reported to be no longer “careworn and feeble looking.” 17

In the fall and winter of 1882, the President developed progressive fatigue, weight loss, loss of appetite, and peripheral edema. While the press continued to report that the President was suffering from malaria, he was evaluated by several physicians for these additional symptoms and was discovered to have evidence of Bright's disease. Bright's disease was diagnosed in the 1880s by evaluating the urine for protein. 18 19 There are no reports from his physicians of a complaint of decreased urine output or dark urine suggestive of oliguria, so it is unclear which symptoms led the physicians to evaluate the President's urine. Since Bright's disease was thought to be a fatal illness in 1882, the President and his physicians decided to keep the suspicion of this illness out of the public domain. In the 1880's, the cause of renal failure was not understood but the only other disease that the President was thought to have that could cause renal failure was malaria.

The diagnosis of renal failure was suggested by a former Surgeon General of the State of New York, Salem H. Wales, and confirmed by an expert from New York City. 20 The name of this expert was never made public but Alfred L Loomis, of the Bellevue Hospital, may have provided an expert opinion for the President concerning the diagnosis of Bright's disease. Loomis consulted on Arthur in 1886 when the former President was in the terminal stages of his kidney failure. It is possible that Arthur relied on Loomis' opinion in 1882 when the diagnosis of Bright's disease was initially made but this cannot be proven by existing records. 21

The President's potentially fatal illness did leak to the press as reported by several sources in October of 1882. 22 23 The Atlanta Constitution reported that the President had “incipient signs of Bright's disease” and the only treatment was the abandonment of his “late hours of night work” and he was suggested to “give closer attention to the laws of health, or suffer the consequences.” 24 Similar reports appear in the St Louis Globe-Democrat on October 9, 25 but the President did not officially respond. The White House attempted to manage the information about the President's health by two stories in the press in October, the first declaring that the Arthur's illness was simply related to a mild case of malaria 26 and the second denying the story about Bright's disease completely. 27

There were several episodes in the next 2 years of his presidency that Arthur was noticeably sick. The most dramatic episode was during his trip to Florida in April of 1883. The main purpose of the trip was rest and relaxation to combat the growing symptoms of fluid retention and energy loss. 28 29 Unfortunately, Arthur had a deterioration of his illness during this trip. The presidential party left Washington on April 5 by train arriving in Jacksonville, Florida, on the evening of April 6. The President then boarded a steamer and traveled via the St Johns River to Sanford, Florida. The President's health started to worsen on the evening of the 8th which did not improve by the morning of the 9th when he traveled to Kissimmee City and felt “savage and dangerous” (as described by his personal secretary William E. Chandler who accompanied the President on this trip). He improved enough to fish in Lake Tohopekaliga, Reedy Creek, and the Kissimmee River for the 9 days and on Wednesday, April 18; he traveled to Savannah on the steamer, the Tallapoosa. On the evening of April 19 on board the Tallapoosa, he developed an episode of colicky abdominal pain, rigors, and nausea. 30 He was seen by the steamer's doctor, Clarence E. Black, who treated him with morphine to manage his pain and allow the President to relax. 31 He improved some but the following morning was described as feeble, ill, feverish, and irritable. He was not feeling well until he returned to Washington, D.C. on the evening of April 22. Chandler denied the “rumors” of the President's ill health and suggested that he had an attack of indigestion. The President was described as having put on weight and claimed that he was not sick a single day during his Florida trip. 32 33 34 35

The illness experienced on his Florida fishing trip was one of several times that the President's symptoms suggested an attack of malaria, although there is no medical proof that this and other episodes that he experienced were in fact malaria. In the 1880s, the diagnosis of malaria was clinical without a definitive diagnostic test. Later in his presidency, he continued to do poorly from a medical point of view. He entertained less and sought out ways to improve his health. He was advised that a trip out West would be beneficial so he took a one-month excursion to Yellowstone National Park. Fortunately, the President's health improved some during this western trip but his renewed vigor was not longlasting. 36

In 1884, as the next presidential campaign approached, Arthur did not withdraw his name from consideration but went into the republican convention in Chicago in June of 1884 without an organized campaign effort. James G. Blaine was the front-runner prior to the convention and secured the nomination on the fourth ballot. Grover Cleveland was the Democratic Party nominee and defeated Blaine capturing 48.9% of the popular vote. Cleveland won 219 electoral votes to 182 for Blaine. 37

## Arthur after the Presidency

Arthur served out his term until March of 1885 without further health difficulties. After leaving office, he refused an effort to draft him into running for the U.S. Senate from New York and instead elected to work with the law firm, “Arthur, Knevals and Ransom.” 38 Due to declining health, he rarely conducted much business with the firm. He spent most of his time at home and often in bed as his renal failure worsened. He lost his appetite and began to lose weight, symptoms associated with uremia, although he did not appear to develop the characteristic mental incapacity associated with the condition. He was seen only occasionally in public in 1885 as his symptoms of weakness worsened. 39

The story of his Bright's disease officially broke in June of 1885 with an article in the New York Times. In that article his loss of appetite was highlighted, as was the fact the he was avoiding public appearances. 40 He was being maintained on a milk-based diet, which was common in the treatment of end-stage Bright's disease. 41 42

The President died on November 18, 1886 at the age of 57. The day before he suffered a major stroke and became comatose, he was declared dead the following day. On November 16, just prior to his stroke, Arthur made the unusual request that all of his personal and official papers be burned, leaving almost no record of his professional life in and out of the White House. There is no known motive for Arthur's directive to destroy his records. Historians have suggested that Arthur was worried about his time as Collector of the Port of New York, his broad support of the patronage system, and the impact of these actions on his legacy. A small volume of presidential papers did survive Arthur's efforts to erase his past but they do not provide a motive for doing so. 43

## Cause of Death

Sources agree on the cause of Arthur's terminal event on November 17 and 18, 1886. The description of a stroke is clear and the doctors that managed his care at this time would have had adequate time to make this diagnosis before he died the following day. His stroke may have resulted from high blood pressure associated with renal failure although this vital sign was rarely measured in the 19th century and there is no documentation that Arthur suffered from hypertension.

## The President's Renal Failure and Potential Causes

By every historical account, the President had Bright's disease and his symptoms support this diagnosis. Given the fact that the diagnosis was easy to confirm in the 1880s and the doctors spoke with relative certainty, it is assumed that the President had renal failure associated with albuminuria, the diagnostic finding in Bright's disease. 44

The cause of the President's renal failure is unknown. By close examination of the President's actions, activities, and exposures, the following are possible causes of the President's renal failure. Poststreptococcal infection syndrome was a common causes of renal failure in the mid-1800s. 45 In the preantibiotic era, streptococcal infections were poorly treated and were either lethal or a cause of major morbidity. Arthur appeared to enjoy excellent heath prior to 1880. There are no references to prolonged illnesses that could be remotely related to a strep infection. Just before the 1880 presidential elections, the President's wife died of pneumonia and it is possible that this was a strep pneumonia. There is no evidence that the President suffered from any illness in the run up to the presidential election. During a presidential election, due to press coverage, serious illness would have been difficult to hide.

Poststreptococcal renal failure is classified as a glomerulonephritis. Other diseases including Goodpasture's syndrome, Wegener's disease, and polyarteritis nodosa may also cause glomerulonephritis. All of these diseases can lead from acute glomerulonephritis to chronic glomerulonephritis and eventually renal failure. All are associated with a syndrome that would start with the specific medical problem with renal failure as a later finding. Rare causes of glomerulonephritis are tumors that produce a paraneoplastic syndrome. These tumors can be solid or hematologic in origin and renal dysfunction maybe the initial manifestation of the malignancy. 46 Arthur had excellent health until 1881, and therefore there are little or no medical clues that would suggest one of the conditions of glomerulonephritis as a cause.

Obstructive uropathy is the least likely cause of renal failure in Arthur. Patients with unilateral kidney obstruction are often very symptomatic and do not usually develop renal failure because they have a functioning kidney. Patients with obstruction at the bladder neck, prostate, or urethra are also symptomatic and there is often urinary infection or significant voiding problems. The management of bladder obstruction would likely have been diagnosed and managed with dilation, which was appropriate for the 1880s. 47 Additionally, patients that have recurrent ascending urinary infection (pyelonephritis) can eventually develop renal failure. There is no evidence that Arthur suffered from recurrent pyelonephritis, a syndrome that was well known in the 1880s.

In the 1880s, exposure to nephrotoxic agents, such as drugs, was not a common cause of renal failure. The President did not appear to be taking any nephrotoxic agents making this cause of renal failure very unlikely.

Poorly controlled diabetes was relatively common in the 19th century and well recognized by organized medicine. Patients characteristically presented with excessive thirst, polyuria, polydipsia, strokes, heart attacks, peripheral vascular disease, and recurrent infections. Complications of diabetes leading to renal failure were common. In the 1880s, the diagnosis of diabetes would have been made by symptoms and glucose in the urine, and therefore it is very unlikely that the President had undiagnosed diabetes. 48

Hypertension can be a cause of renal failure and can result from renal failure. The syndrome of hypertensive diseases often includes headaches, nosebleeds, cardiac disease, strokes, and peripheral vascular disease. There is no historical evidence that the President suffered from any of these symptoms. It would be unusual for primary hypertension to present initially as renal failure without the other manifestations of the disease.

There are varieties of autoimmune diseases (immunoglobulin A [IgA] nephropathy, Sjögren syndrome, systemic scleroderma, autoimmune myopathies, systemic lupus erythematosus, antiphospholipid syndrome nephropathy, and rheumatoid arthritis) that are associated with various degrees of renal failure. It would be rare for the renal failure to be the presenting symptom in the autoimmune disorders. Invariably, the other systemic manifestations are the presenting symptoms and the renal failure is a late finding. 49

## Malaria

It is likely that the President also suffered from malaria. When President Arthur became sick in 1881, it was reported that he had malaria. Some historians have assumed that the news stories about malaria were simply a cover story for the truth about his terminal Bright's disease. 50 Although Arthur spent much of his adult life in New York City, and in other cities that were very low risk for malaria, in Washington, D.C. in the 1880s malaria was very common. Several famous Washington, D.C. politicians suffered from malaria including George Washington. 51 President Arthur was well known to take walks during his presidency, usually late at night after midnight. These evening walks around the city provided daily exposure to the disease. The attack he experienced in Florida in 1883 had symptoms that were similar to a severe exacerbation of malaria especially the colicky pain associated with rigors. In the 1880s, malaria was a chronic relapsing illness despite treatment. 52

The modern treatment of malaria has been well established, 53 but the usual treatment of malaria in the 1880s was quinine. 54 55 Quinine was effective at clearing the blood of the parasite and often resulted in remission. It was also common that patients would relapse with recurrent symptoms due to reinfection or by failure of the quinine to eradicate the parasite in the liver. Several other drugs were used to treat malaria in the 19th century such at arsenic, iodine, and carbolic acid. Of the available treatments, quinine was the most effective with the least side effects. 56 Although news reports often mentioned the President's malaria, there is no mention of its treatment or his response to treatment.

Renal failure is one of the complications of malaria that would have shortened the President's life. Acute renal failure has been described with the initial episode of malaria or during an exacerbation. Some patients improve after this episode of acute renal failure and recover their kidney function; others go on to develop chronic renal insufficiency and eventually renal failure. 57 58 Based on the available information it is impossible to determine the cause of the President's renal failure but malaria a significant possibility.

## Conclusion

Based on the evidence that is available in the press reports of the time, papers that were preserved by Arthur's relatives, and the documents available from his medical doctors, President Chester A. Arthur appears to have acquired malaria early during his tenure as vice president or president while living in Washington, D.C. In addition, he developed Bright's disease sometime in 1881. He had a significant downhill clinical course after his presidency and died the day after a severe stroke. The cause of his renal failure is unknown.
